# Fabrication and In Vitro Evaluation of pH-Sensitive Polymeric Hydrogels as Controlled Release Carriers

**DOI:** 10.3390/gels7030110

**Published:** 2021-08-05

**Authors:** Muhammad Suhail, Chih-Wun Fang, Arshad Khan, Muhammad Usman Minhas, Pao-Chu Wu

**Affiliations:** 1 School of Pharmacy, Kaohsiung Medical University, 100 Shih-Chuan 1st Road, Kaohsiung City 80708, Taiwan; Suhailpharmacist26@gmail.com; 2 Divison of Pharmacy, Zuoying Branch of Kaohsiung Armed Forces General Hospital, Kaohsiung City 81342, Taiwan; u101530009@gap.kmu.edu.tw; 3 Department of Pharmaceutics, Faculty of Pharmacy, Khawaja Fareed Campus (Railway Road), The Islamia University of Bahawalpur, Bahawalpur 63100, Pakistan; arshadpharma77@gmail.com; 4 College of Pharmacy, University of Sargodha, Sargodha 40100, Pakistan; 5 Department of Medical Research, Kaohsiung Medical University Hospital, Kaohsiung 80708, Taiwan; 6 Drug Development and Value Creation Research Center, Kaohsiung Medical University, Kaohsiung 80708, Taiwan

**Keywords:** hydrogels, swelling test, drug release

## Abstract

The purpose of the current investigation was to develop chondroitin sulfate/carbopol-co-poly(acrylic acid) (CS/CBP-co-PAA) hydrogels for controlled delivery of diclofenac sodium (DS). Different concentrations of polymers chondroitin sulfate (CS), carbopol 934 (CBP), and monomer acrylic acid (AA) were cross-linked by ethylene glycol dimethylacrylate (EGDMA) in the presence of ammonium peroxodisulfate (APS) (initiator). The fabricated hydrogels were characterized for further experiments. Characterizations such as Scanning electron microscopy (SEM), Thermogravimetric analysis (TGA), Differential scanning calorimetry (DSC), Powder X-ray diffractometry (PXRD), and Fourier transform infrared spectroscopy (FTIR) were conducted to understand the surface morphology, thermodynamic stability, crystallinity of the drug, ingredients, and developed hydrogels. The swelling and drug release studies were conducted at two different pH mediums (pH 1.2 and 7.4), and pH-dependent swelling and drug release was shown due to the presence of functional groups of both polymers and monomers; hence, greater swelling and drug release was observed at the higher pH (pH 7.4). The percent drug release of the developed system and commercially available product cataflam was compared and high controlled release of the drug from the developed system was observed at both low and high pH. The mechanism of drug release from the hydrogels followed Korsmeyer–Peppas model. Conclusively, the current research work demonstrated that the prepared hydrogel could be considered as a suitable candidate for controlled delivery of diclofenac sodium.

## 1. Introduction

Hydrogels are three-dimensional network structures having the ability to absorb a large amount of water or physiological medium due to the presence of hydrophilic polymers, which possess a large number of hydrophilic groups [1,2,3]. Hydrogels can hold a high amount of water and cannot be dissolved by water due to the strong linkages (physically or chemically) that formed between the functional groups of the polymer’s chains. Both synthetic and natural polymers and their combination are employed for the development of hydrogels by various crosslinking methods [4,5]. Hydrogels resemble living tissues in swelled form indicating their rubbery and soft nature. The encapsulated drug is protected by hydrogels from the unfriendly environment of the body [2,6]. The structural and morphological responses of hydrogels—swelling/deswelling, sol–gel transition, or degradation—are altered with the environmental changes. These environmental stimuli can be physical and chemical, such as chemical agents [7], light [8,9], temperature [10,11], and pH [12,13]. Stimuli-responsive hydrogels have been widely considered as drug-controlled carriers due to their unique characteristics, i.e., swelling/deswelling responses and sol–gel transition [13,14]. In the case of semi-interpenetrating polymer network techniques, different polymers are combined in a such way that one polymer is crosslinked only and persists there without generating any further non-covalent interaction amid two polymers [15]. The semi-interpenetrating hydrogels possess intermediary mechanical and physical properties of both polymers contributing in the reaction. When polymers with high mechanical strength and low water swellability are crosslinked with polymers of low mechanical strength and high swellability, semi-interpenetrating hydrogel’s structures with intermediary features are generated as a result. The semi-interpenetrating techniques help mostly to improve the mechanical and physical properties of such hydrogels that are based on biodegradable natural polymer [16]. Recently, this technique has been employed by a number of researchers to improve the mechanical and physical properties of the biodegradable natural polymer-based hydrogels [17,18].

Chondroitin sulfate (CS), a natural polymer obtained from animal cartilage, is used in the development of various dosage forms to treat different diseases. Chemically, CS contains D-glucuronic acid interconnected to *N*-acetyl-d-galactosamide [19]. Connective tissues are protected by CS by means of regulating its water content and therefore used for a long duration to treat serious disorders such as atherosclerosis, thrombosis, and osteoarthritis [20,21]. CS is considered as a potential candidate for the absorption of a high quantity of water by the hydrogels network due to the presence of active functional groups such as COO− and –SO_3_− [22]. Carbopol 934 (CBP) polymers are known as GRAS (generally regarded as safe) materials and used mostly in oral drug delivery system such as capsules, tablets, and suspensions, and in topical drug delivery systems [23]. Due to their diverse characteristics, this type of polymer is used frequently for biomaterial delivery. Due to its viscous nature, CBP is used mostly in the preparation of various types of gels compared to natural gums, because the thickening ability of CBP is higher in both organic solvent and aqueous solution as compared natural gums [24,25]. As the concentration of CBP increases, the formation of fusion complex of the formulation increases. CBP is hydrophilic in nature and its COOH groups ionized highly after neutralization and resulted in gel formation due to the electrostatic repulsion of the same charged polymer chains. Due to its low toxicity, high compatibility, and stability, CBP is frequently used in cosmetic and pharmaceutical industries [26,27]. Acrylic acid (AA) is a synthetic monomer. It is pH-sensitive by nature. The swelling index of AA is greatly reliant on the surrounding (pH) medium. AA contains COOH groups which are highly linked with the molecules of water; thus, equilibrium swelling is affected largely by pH and ionic strength of the stated medium [28]. The pH-sensitive nature enables AA to fabricate pH-sensitive hydrogels and thus plays an important role in sustained, controlled, and targeted drug delivery systems [6,29].

Diclofenac sodium (DS) is a nonsteroidal anti-inflammatory drug (NSAID) that is available commercially in its salt form such as sodium or potassium [30]. The half-life of DS is 2 h [31,32]. Orally administered DS is absorbed very rapidly and completely. Available commercial dosage forms of DS are as tablets, capsules, and topical gels. The available dose of DS as a tablet is 25, 50, 75, and 100 mg, while as a capsule it is 18 and 35 mg, respectively, taken multiple times a day. A repeated dose of DS leads to some complications such as the formation of gastrointestinal lesions and kidney damage [33,34,35]. Hence, to overwhelm the problem concerned with rapid administration and enhance the patient compliance, some new strategies are required. Therefore, the authors prepared CS/CBP-co-PAA hydrogels to prolong the drug release in a controlled way.

The objective of the present research was to develop the pH-sensitive polymeric network of hydrogels via a free-radical polymerization technique for controlled delivery of DS. Such a polymeric network may be employed to overcome the problems associated with the conventional systems and may assist to keep the drug steady state plasma concentration, decreasing the frequency of dose due to greater loading capability and ultimately enhanced patient compliance. A series of nine formulations was carried out for the evaluation and analysis of CS/CBP-co-PAA hydrogels. CS and CBP were used as natural and synthetic polymers, whereas AA and EGDMA were used as monomer and cross-linker, respectively. Several experiments such as sol–gel analysis, swelling index, drug loading, in vitro drug release and kinetic modelling were conducted. Along with this, characterizations such as SEM, TGA and DSC, PXRD, and FTIR were carried out to assess and analyze the various features of the fabricated hydrogels.

## 2. Results and Discussion

### 2.1. Morphological Analysis (SEM)

**Figure 1:**
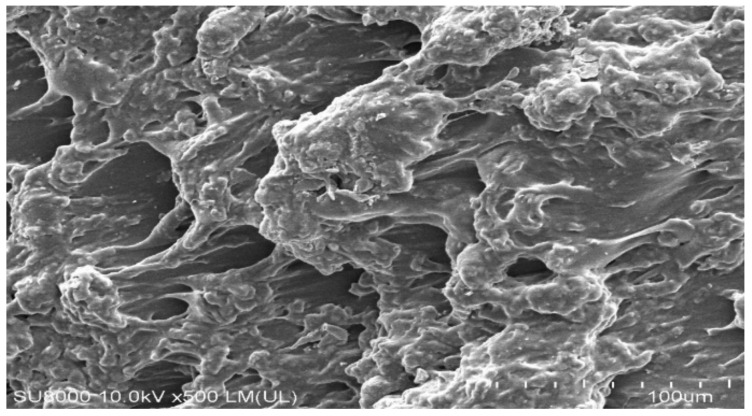
Morphological analysis of CS/CBP-co-PAA hydrogels.

Morphological analysis was performed by SEM for the purpose of evaluating the structural morphology of CS/CBP-co-PAA hydrogels (Figure 1). A highly porous structure is revealed by the developed hydrogels, which leads to maximum dynamic swelling, loading of drug, and drug release from the developed hydrogels [36].

### 2.2. TGA Measurement

**Figure 2:**
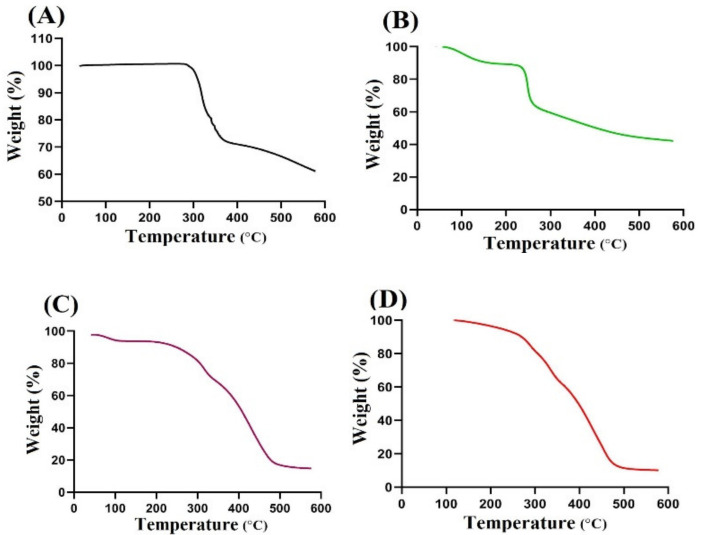
TGA (Thermogravimetric analysis) of (**A**) DS (Diclofenac sodium), (**B**) CS (Chondroitin sulfate), (**C**) CBP (Carbopol), and (**D**) CS/CBP-co-PAA hydrogels.

A TGA thermogram of DS, CS, CBP, and CS/CBP-co-PAA hydrogels is shown in Figure 2A–D. At three different stages, weight loss is shown by TGA of DS (Figure 2A). At the first stage, a 26% reduction in weight is observed within a temperature range of 325–368 °C tracked by dehydration. At the second stage, a 20% reduction in weight is observed as the temperature approaches 464 °C. At the final stage, drug pyrolysis is started at 470 °C till entirely paralyzed [37]. Similarly, weight reduction of CS takes place at three different stages (Figure 2B). The first stage initiates from 105 °C to 258 °C and a 45% reduction in weight is observed due to moisture loss of polymer chains. Onward, a 15% weight reduction is observed within a temperature range of 260 °C to 361 °C. Weight reduction may reveal the initial degradation of carboxylate and sulfonate groups of the CS at this points, whereas at the last stage, degradation of CS initiates from 367 °C up to entire degradation [38]. As with DS and CS, weight reduction is assigned by TGA of CBP (Figure 2C) at three different stages. Initially, a 13% weight reduction is revealed at 103 °C tracked by moisture loss; after this, a 26% reduction in weight of CBP is detected due to decarboxylation, development of unsaturated structures, and depolymerization of the polymer as the temperature approaches 320 °C, and at final stage, degradation of CBP starts from 410 °C until entirely paralyzed [39]. A TGA thermogram of CS/CBP-co-PAA hydrogel is shown in Figure 2D. A TGA thermogram of the developed hydrogel reveals a higher degradation half-life (t_1/2_ = 480 °C) compared to the degradation half-lives of two polymers, i.e., CS (t_1/2_ = 376 °C) and CBP (t_1/2_ = 410 °C), respectively, indicating its higher thermal stability. The TGA of CS/CBP-co-pAA hydrogels indicates a weight reduction of 15% from 200 °C to 280 °C. After that, a 65% reduction in weight is exhibited between 280 °C to 480 °C due to the breakdown of functional groups of polymers, i.e., sulfonate and carboxylate groups. Further degradation of fabricated hydrogels is started from 480 °C until entire pyrolysis. Hence, the above results indicate that CS/CBP-co-PAA hydrogel is thermally more stable than its contents, i.e., polymers (CS and CBP).

### 2.3. DSC Measurement

**Figure 3:**
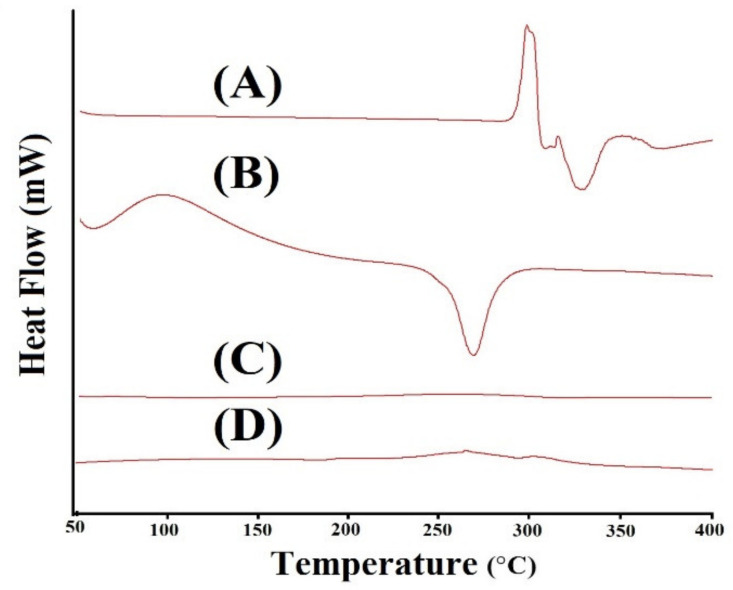
DSC (Differential scanning calorimetry) of (**A**) DS (Diclofenac sodium), (**B**) CS (Chondroitin sulfate), (**C**) CBP (Carbopol), and (**D**) CS/CBP-co-PAA hydrogels.

Figure 3A–D indicates DSC of DS, CS, CBP and CS/CBP-co-PAA hydrogel. DSC thermogram of DS (Figure 3A) assigns two endothermic peaks at 288 °C and 330 °C, correspondingly. Similarly, two exothermic peaks at 298 °C and 355 °C are perceived, assigned to the glass transition temperature and drug degradation [40]. An endothermic peak is shown by DSC thermogram of CS (Figure 3B) within a temperature range of 55–76 °C tracked by dehydration and elimination of other volatile constituents. Degradation of polymer chains is observed by a prominent endothermic peak at 263 °C. Two exothermic peaks were detected at 104 °C and 274 °C led to glass transition temperature and oxidative degradation of the polymer [41]. DSC of CBP (Figure 3C) reveals two endothermic peaks at 68 °C and 236 °C. The endothermic peak at 68 °C may be due to evaporation of unbound water present in CBP, whereas the peak at 236°C may be due to the anhydrides formation in the polymer chain [42]. Similarly, two exothermic peaks at 96 °C and 228 °C are observed [43]. Figure 3D indicates the DSC thermogram of CS/CBP-co-PAA hydrogels. An endothermic peak is perceived at 270 °C, indicating that the endothermic peak of CS shifted from 263 °C to 270 °C in CS/CBP-co-PAA hydrogels, representing greater stability and constancy of fabricated hydrogels. Similarly, two exothermic peaks at 280 °C and 320 °C are observed, which are the exothermic peaks of CS and CBP moved from 274 °C and 228 °C to 280 °C and 320 °C, which led to glass transition and oxidative degradation of the developed hydrogels. These all indicate the higher thermal stability of the developed hydrogels. B. Singh et al. prepared antibiotic drug-loaded hydrogel dressings for better wound management, and reported higher thermal stability for the developed system [44].

### 2.4. PXRD Analysis

**Figure 4:**
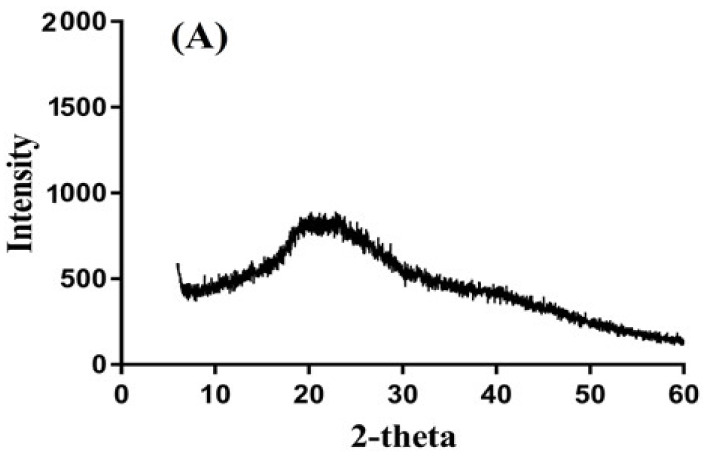

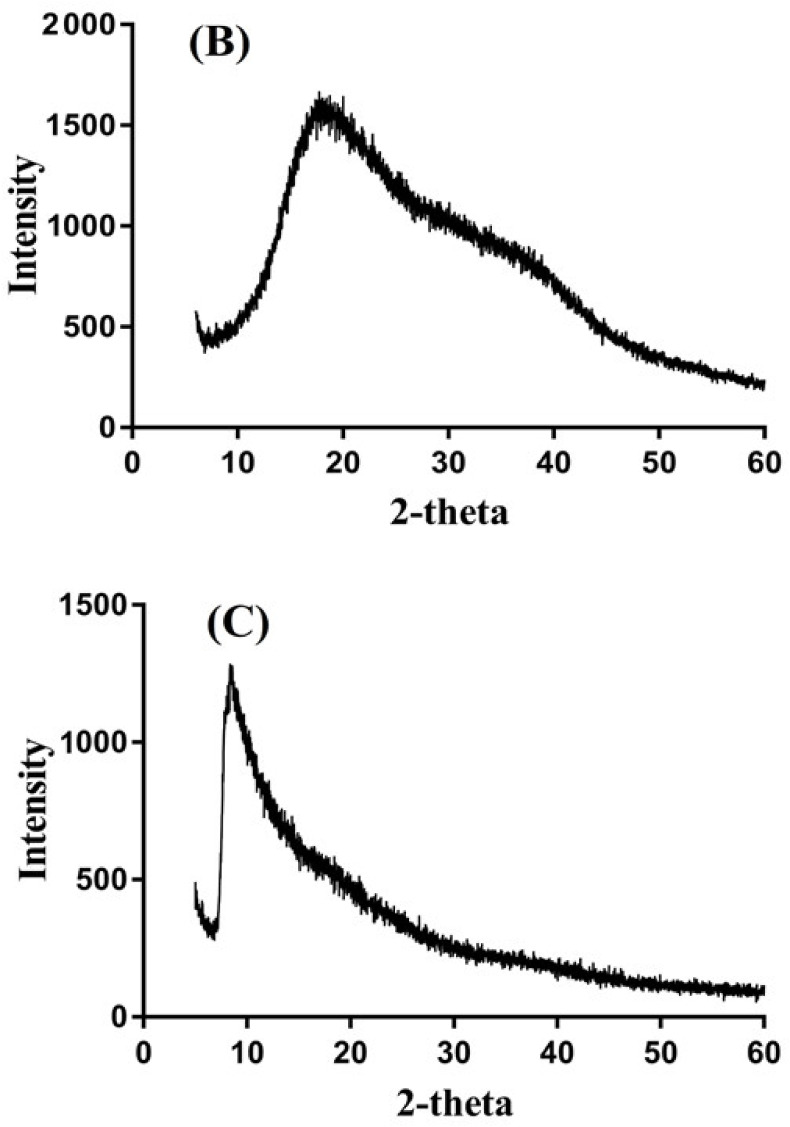

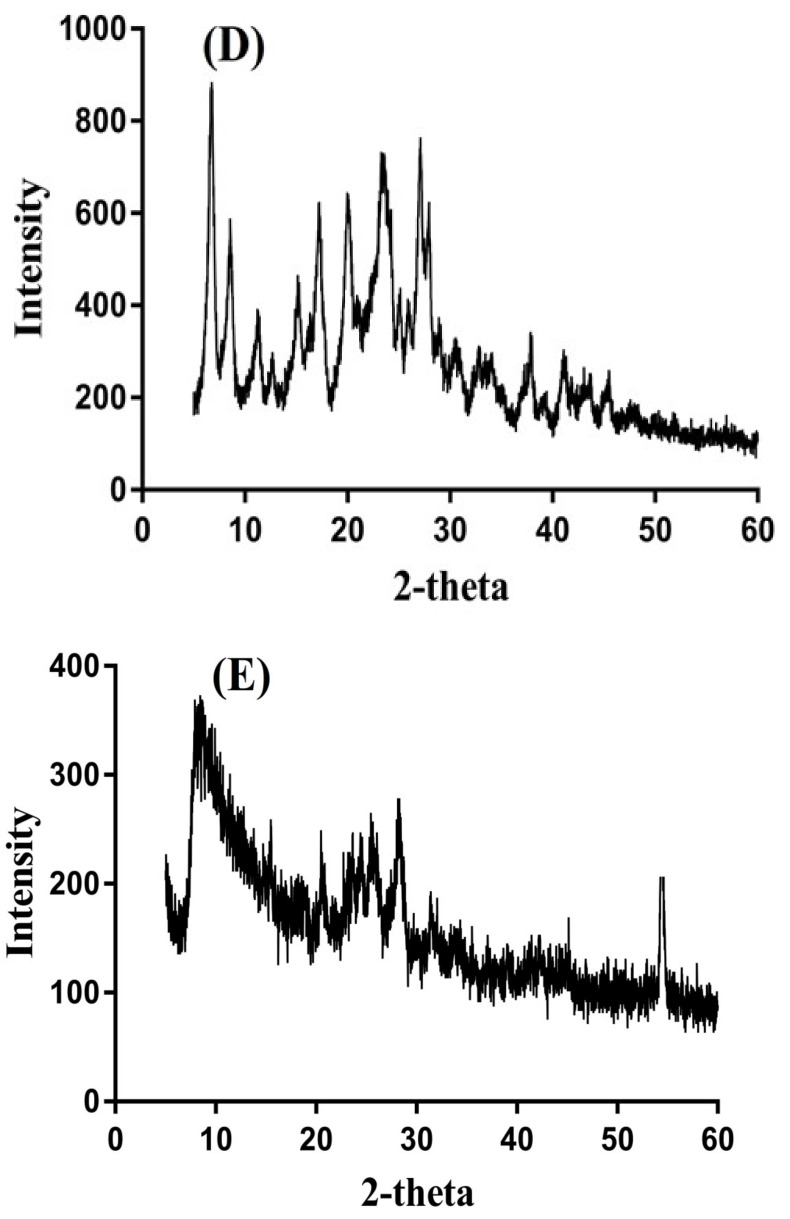
PXRD (Powder X-ray diffractometry) of (**A**) CS (Chondroitin sulfate), (**B**) CBP (Carbopol), (**C**) unloaded CS/CBP-co-PAA hydrogels, (**D**) DS (Diclofenac sodium), and (**E**) drug-loaded CS/CBP-co-PAA hydrogels.

PXRD analysis of CS, CBP, unloaded CS/CBP-co-PAA hydrogels, DS, and drug-loaded CS/CBP-co-PAA hydrogels is shown in Figure 4A–E. PXRD of CS (Figure 4A) reveals some small crystalline peaks at 2θ = 20.43°, 22.76°, and 27.16. Similarly, PXRD of CBP (Figure 4B) reveals peaks at 2θ = 19.63°, 23.23°, and 32.73°, respectively, which determine its crystalline nature. The small crystalline peaks of both the polymers are disappeared, as indicated by the PXRD of unloaded CS/CBP-co-PAA hydrogels (Figure 4C). The reason is the high crosslinking of CS and CBP with AA during polymerization reaction; hence, an amorphous network of hydrogels is developed. The high-intensity peaks of DS are detected at 2θ = 22.30°, 24.64°, 27.63°, and 38.90°, which indicate the crystalline nature of the drug (Figure 4D). The intensity of crystalline peaks of the drug is reduced as drug is encapsulated by the polymeric system. The intensity of the above-mentioned peaks of DS is reduced as the drug is loaded by CS/CBP-co-PAA hydrogels (Figure 4E), which reveals the successful encapsulation and reduction of DS crystallinity by the fabricated hydrogels. Sarfraz et al. also reported the same results for carbopol-based hydrogel nanoparticles, which further supports our studies [45].

### 2.5. Measurement of FTIR

**Figure 5:**
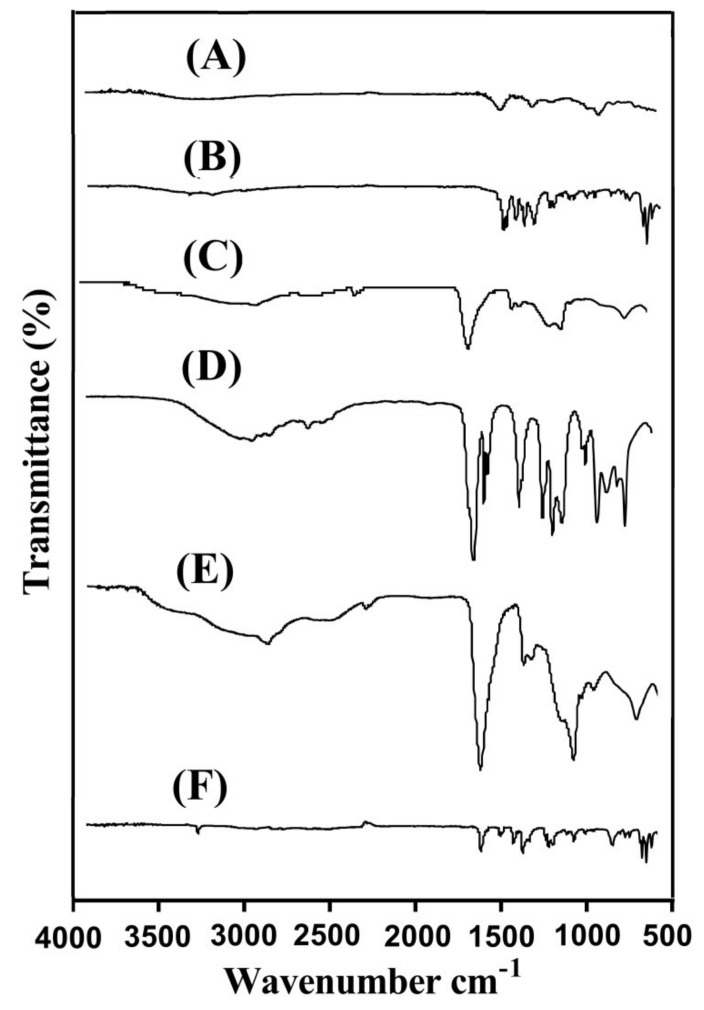
FTIR (Fourier transform infrared spectroscopy) spectra of (**A**) DS (Diclofenac sodium), (**B**) CS (Chondroitin sulfate), (**C**) CBP (Carbopol), (**D**) AA (Acrylic acid), (**E**) unloaded CS/CBP-co-PAA hydrogels, and (**F**) loaded CS/CBP-co-PAA hydrogels.

FTIR is analyzed for DS, CS, CBP, AA, unloaded CS/CBP-co-PAA hydrogels, and drug-loaded CS/CBP-co-PAA hydrogels, as shown in Figure 5A–E. The distinctive band of DS is perceived at 3336 cm^−1^, revealing stretching vibration of COOH (Figure 5A), whereas stretching vibrations of N─H and C=C are detected at 3398 and 1593 cm^−1^ [46,47]. Similarly, the FTIR spectrum of CS indicates a prominent peak at 1560 cm^−1^ assigning the stretching vibration of amide group (Figure 5B). A broad peak is detected at 3410 cm^−1^ due to –OH stretching vibration. The presence of carboxyl group is confirmed by the overlapping of two different functional groups, i.e., ─OH and C─O at 1430 cm^−1^ and 1387 cm^1^ respectively. Likewise, S=O stretching vibration of the sulfate group is detected at peak 1250 cm^−1^ [48,49]. Similarly, CBP FTIR spectra (Figure 5C) reveal stretching vibration of C=O, OH, and R-CH_2_ at 1632, 2703, and 2998 cm^−1^, respectively [50,51,52]. Stretching vibration of ─C─C and ─CH_2_ groups of the AA (Figure 5D) assign prominent peaks at 1520 and 3010 cm^−1^, while the prominent bands of –C–O–C and –C=O are assigned at 1090 and 1312 cm^−1^, respectively [53,54]. The FTIR spectra of unloaded CS/CBP-co-PAA hydrogels are shown in (Figure 5E). The characteristic peaks of CS, CBP, and AA are shifted to developed unloaded hydrogels due to the electrostatic interaction among the various functional groups of CS, CBP, and AA. The specific –OH group of CS at peak 3410 cm^−1^, R-CH_2_ group of CBP at peak 2998 cm^−1^ and H-C-H group of AA at peak 3010 cm^−1^ are overlapped by a peak at 3210 cm^−1^ of unloaded developed hydrogels. Some new peaks are observed in unloaded CS/CBP-co-PAA hydrogels. The shifting and formation of new peaks reveal that AA is grafted completely over the backbone of CS and CBP. Likewise, some peaks of the drug such as 3336 and 1593 cm^−1^ are moved to 3362 and 1598 cm^−1^ peaks of the loaded CS/CBP-co-PAA hydrogels (Figure 5F), that reveal the successful loading of the drug by the developed hydrogels and no interaction between the drug and hydrogel ingredients is detected [55].

### 2.6. Influence of pH and Composition on Swelling Behaviors

**Figure 6:**
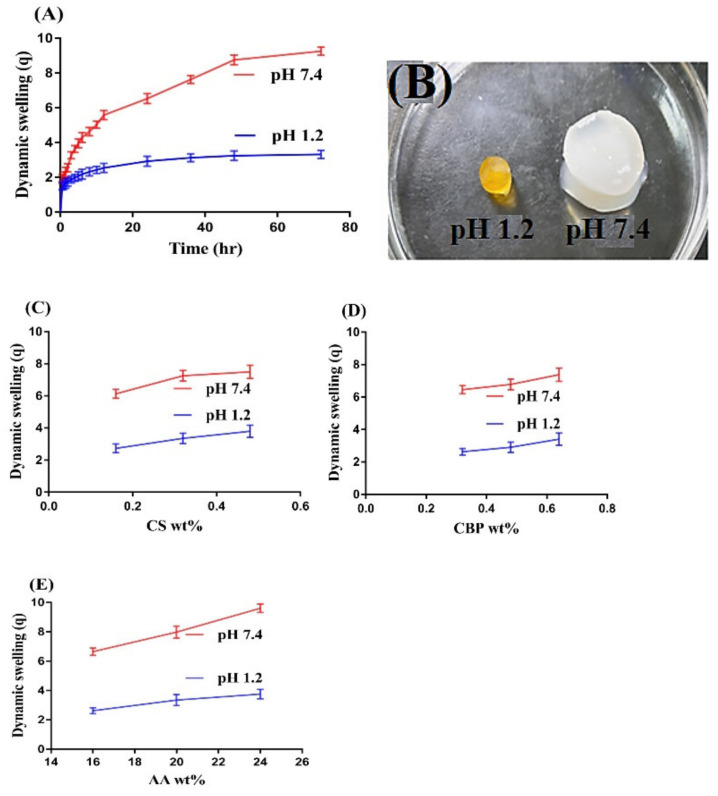
(**A**) Effect of pH on swelling dynamics; (**B**) swelled hydrogel at pH 1.2 and 7.4; effect of (**C**) CS (Chondroitin sulfate), (**D**) CBP (Carbopol), and (**E**) AA (Acrylic acid) on dynamic swelling of CS/CBP-co-PAA hydrogels.

Dynamic swelling is conducted for CS/CBP-co-PAA hydrogels at both pH 1.2 and pH 7.4, as shown in Figure 6A,B. The pH greatly affects the swelling index of the developed hydrogel network as dynamic swelling is greater at pH 7.4 compared to pH 1.2. The main reason is the presence of COOH groups of CS, CBP, and AA, that increase as the pH of the system increases. At low pH, protonation of COOH groups takes place that conjugate with other counter ions, hydrogen bonding interaction occurs between COOH groups and as a result low dynamic swelling is observed at pH 1.2. Whereas as the pH of the medium enhances, deprotonation of COOH groups takes place, more free COOH groups are generated and interaction of hydrogen bonding is decreased. These free COOH groups exert electrostatic repulsive forces; as a result, greater dynamic swelling is detected at pH 7.4 [56]. Similarly, the composition of CS, CBP, and AA also influences the dynamic swelling of the developed hydrogels, as shown in Figure 6C–E. At a constant composition of CBP and AA, when the composition of CS (Figure 6C) increases, a rise in swelling index is observed at both pH 1.2 and 7.4. The reason for higher swelling is the presence of COOH, SO_3_, and OH groups of the CS that enable this polymer to interact highly with the water and show its hydrophilic properties. These functional groups of CS exert electrostatic repulsive forces and as a result the volume of developed system increases and swelling increases [56]. Similarly, as the composition of CBP (Figure 6D) increases at a constant composition of CS and AA, increase in the swelling index is observed at both pH 1.2 and 7.4. The reason is the generation of greater COOH groups and vice versa [57]. Like CS and CBP, the swelling index increases at both pH 1.2 and 7.4 as the composition of the AA (Figure 6E) increases at constant composition of CS and CBP. The pKa value of AA is 4.2; hence, chains of AA are collapsed at pH 1.2, and as a result, a low swelling index is detected at pH 1.2. However, as the pH increases above 6 and 8, carboxylate ions are generated, which cause the repulsion of chains and greater swelling is exhibited [58].

### 2.7. Sol–Gel Measurements

Sol–gel measurements were conducted for CS/CBP-co-PAA hydrogels to analyze the sol and gel fraction of the fabricated hydrogels network. Gel fraction for all formulations (CCF-1 to CCF-9) is found to be 85~96%, whereas sol fraction is 15~4%. As CS concentration (CCF-1-CCF-2 and CCF-3) is increased while keeping the concentration of CBP and AA constant, gel fraction (89~94%) is increased. The higher gelation is because of greater availability of reactive sites for the monomer contents during polymerization reaction; thus, as the CS concentration increases, gel fraction increases [50]. Similarly, the number of reactive sites increases for polymerization of monomer contents as the concentration of CBP (CCF-4, CCF-5, and CCF-6) increases at a constant composition of CS and AA; hence, a rise in gel fraction (87~94%) is observed [59]. Like CS and CBP, an increase in gel fraction (85~96%) is observed with the increase in the concentration of AA (CCF-7, CCF-8, and CFF-9) while other contents remain constant. The reason is the availability of higher amount of reactive site of the polymers, so the greater the reactive sites, the higher the polymerization process will be among the hydrogels contents, and the maximum will be the gelation, and vice versa [60]. Decrease in sol fraction is observed as the concentration of the CS, CBP, and AA increases because sol fraction is inversely proportional to gel fraction [61].

### 2.8. Loading of Drug

Quantification of drug loaded by CS/CBP-co-PAA hydrogels is performed for the purpose to know the quantity of the drug encapsulated by the fabricated hydrogels as shown in Table 1. As the composition of CS (CCF-1, CCF-2, and CCF-3) increases, increase in drug loading is observed. Similarly, drug loading is increased by increasing the composition of CBP (CCF-4, CCF-5, and CCF-6). Like CS and CBP, as the composition of AA (CCF-7, CCF-8, and CCF-9) increases, a rise in drug loading is observed. Drug loading is directly related to the hydrogels swelling. The greater the dynamic swelling, the higher the drug loading will be, and vice versa [62].

### 2.9. In Vitro Drug Release Analysis

**Figure 7:**
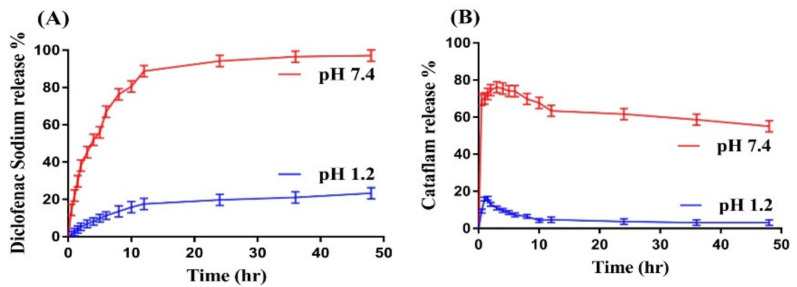
Effect of pH on percent drug release from (**A**) CS/CBP-co-PAA hydrogels and (**B**) commercial product Cataflam.

A release study is carried out for all formulations of CS/CBP-co-PAA hydrogels and commercial product Cataflam at both pH 1.2 and pH 7.4, correspondingly. Greater percent drug release from the developed network is perceived at pH 7.4 as compared to pH 1.2, as indicated in Figure 7A. The developed hydrogel networks exhibit pH-dependent swelling, and the maximum swelling is exhibited at pH 7.4. Similarly, pH-dependent drug release is observed for the developed hydrogels due to pH-dependent swelling of hydrogels. Due to deprotonation of carboxylic groups of CS and CBP and generation of carboxylate ions by AA in greater quantity at high pH, maximum swelling and high percent drug release is detected at pH 7.4. Hence, pH-dependent drug release is shown by all formulations of the fabricated hydrogels [63]. Figure 7B indicates the percent release of Cataflam. A high percent release of drug (77%) is observed within 2 to 3 h at higher pH 7.4, whereas a low percent drug release (17%) is exhibited within 1 to 2 h at lower pH 1.2. After that, a drop is seen in percent drug release at both pH 1.2 and 7.4, respectively. An increase in the concentration of CS (CCF-1, CCF-2, and CCF-3) results in an increase in the percent drug release (Table 1) while keeping the constant concentration of CBP and AA. The escalation in percent drug release is because of the deprotonation of COOH groups and sulfonic group [64]. A slight drop in percent drug release from the CS/CBP-co-PAA hydrogels is detected as the CBP concentration (CCF-4, CCF-5, and CCF-6) is increased, as shown in Table 1. Due to the formation of tight hard gel, a decline in percent drug release is seen. The main cause of reduction in the percent drug release is the high viscosity of the hydrogel networks. The system viscosity is increased as drug is penetrated into hydrogels; thus, the drug is not released easily from the high viscous system [65]. Khan and Zhu et al. also reported the same result, which further supports our studies [66]. Like CS, as the concentration of AA (CCF-7, CCF-8, and CCF-9) increases, a rise in drug release is observed at a constant concentration of other contents (Table 1) [67]. Comparing the percent drug release of commercial product and the developed system, we can conclude that the fabricated system has prolonged the high percent release of DS successfully in a controlled way for 48 h. All the formulations of fabricated hydrogels (CCF-1- CCF-9) exhibit the Korsmeyer–Peppas kinetic model. “r” values for all formulation are found within a range of 0.9772–0.9954. The diffusion mechanism, i.e., Fickian diffusion mechanism and non-Fickian diffusion mechanism or anomalous, is determined by the n value. If the n value is equal to 0.5, it shows that the diffusion mechanism is Fickian, whereas if the n value is greater than 0.5, it indicates non-Fickian diffusion or anomalous. n values are found in the range of 0.5213–0.7304 for all formulations, confirming non-Fickian or anomalous diffusion (Table 1) [68,69].

## 3. Materials and Methods

### 3.1. Materials

Diclofenac sodium (DS) was acquired from ALFA-AESAR (Haverhill, MA, USA). Chondroitin sulfate (CS) and carbopol934 (CBP) were obtained from SIGMA-ALDRICH (St. Louis, MO, USA) and Noveon, Inc (Cleveland, OH, USA). Acrylic acid (AA), ammonium peroxodisulfate (APS) and ethylene glycol dimethylacrylate (EDGMA) were purchased from ACROS (Morris Plains, NJ, USA), SHOWA (Osaka, Japan), and ALFA-AESAR (Haverhill, MA, USA), respectively.

### 3.2. Synthesis of CS/CAR-co-PAA Hydrogels

Chondroitin sulfate/carbopol-co-poly(acrylic acid) (CS/CBP-co-PAA) hydrogels were synthesized successfully by a free-radical polymerization technique. CS and CBP were used as polymers, AA as a monomer, and ammonium peroxodisulfate (APS) as an initiator, whereas ethylene glycol dimethacrylate (EGDMA) was used as a cross-linker, respectively. Weighed quantities of all contents were taken separately. CS and CBP solutions were formed by taking accurate quantities of CS and CBP in separate beakers and dissolving them in a required volume of distilled water with continuous stirring at 50 °C. The solution of APS was added into CBP solution. After a proper mixing of polymer and initiator solution, the mixture was added into continuous stirring solution of CS, and then AA was added drop wise into the respective mixture. After that, cross-linker EGDMA was added slowly into the mixture to crosslink the polymers with monomer at their proper sites. The transparent mixture was purged by nitrogen gas to remove any dissolved oxygen, then poured into the glass molds; the mixture was then placed in a water bath at 55 °C for 2 h, and then 65 °C for 20 h. The formed gel was separated from the glass molds, cut into 8 mm pieces, and washed with an ethanol and water (50:50) mixture to remove any soluble unreacted non-crosslinked content of hydrogel discs. All hydrogel discs were dried initially for 24 h at room temperature, and then placed in a vacuum oven at 40 °C for 7 days. The chemical compositions of all the contents of the developed hydrogels are shown in Table 2.

### 3.3. Morphological Analysis

Scanning electron microscope (SEM) (JSM-5300, Tokyo, Japan) was carried out for evaluation of structural morphologies of the CS/CBP-co-PAA hydrogels. Dried discs of hydrogel were cut into the desired particle size and the sample was mounted on an aluminum stub by the help of double adhesive tape. A gold sputter coater was used for coating stubs with gold beneath argon atmosphere. Photomicrographs were used for the surface analysis after scanning of the samples [70,71].

### 3.4. TGA and DSC Measurement

Thermogravimetric analysis (TGA) was carried out to analyze the thermal stability of DS, CS, CBP, and CS/CBP-co-PAA hydrogels as temperature increases. TA instruments Q5000 series Thermal Analysis System (TA instruments, West Sussex, UK) was used for the analysis of all the samples beneath nitrogen purge. Heating was kept for all the samples from 40 to 600 °C. Differential scanning calorimetry (DSC) analysis was conducted to examine the heat of fusion of the DS, CS, CBP, and CS/CBP-co-PAA hydrogels using a PerkinElmer DSC 4000 (PerkinElmer Ltd., Buckinghamshire, UK). The rate of heating was kept at 20 °C/min beneath a nitrogen gas from 50 to 400 °C [72,73].

### 3.5. Powder X- ray Diffractometry (PXRD) Analysis

PXRD (XRD-6000 Shimazu X-ray diffractometer) was used for the analysis of CS, CBP, unloaded CS/CBP-co-PAA hydrogels, DS, and drug-loaded CS/CBP-co-PAA hydrogels at room temperature. A plastic sample holder and glass slide were used for holding and leveling of samples surface. Theta (θ) was kept between 10° to 60° at a rate of 2° 2θ/min at room temperature for the sample analysis [74,75].

### 3.6. Measurement of FTIR

Fourier transform infrared spectroscopy spectrum (Nicolet 380, Thermo Fisher Scientific, MA, USA) was used for the determination of certain functional groups of DS, CS, CBP, AA, unloaded CS/CBP-co-PAA hydrogels and loaded CS/CBP-co-PAA hydrogels by using attenuated total reflectance (ATR) technology. All the samples were crushed into the required particle size and then measured in a range of 4000–500 cm^−1^ [76,77].

### 3.7. Dynamic Swelling Behaviors

Dynamic swelling was performed for all formulations of the hydrogel to assess the effect of pH on the sensitive nature of the developed hydrogels. A weighed quantity of dried hydrogels discs (S1) was immersed in both acidic and basic media (pH 1.2 and pH 7.4) at 37 °C. Swollen discs of hydrogel (S2) were taken out at various time intervals until no change in weight with time was detected [78,79]. Dynamic swelling was calculated by the following equations:

(1) $$\left(q\right)=\frac{{\;S}_{2}}{{\;S}_{1}}$$

where; q = dynamic swelling, S_1_ = initial weight of dried hydrogel disc, and S_2_ = final weight of swollen hydrogel disc at time t.

(2) $$\left(\mathrm{SR}\%\right)=\frac{W_{1}-W_{2}}{W_{2}}\times 100$$

where; W_1_ = weight of swollen hydrogels discs, while W_2_ = weight of dry hydrogel discs.

### 3.8. Sol–Gel Measurements

Sol–gel fraction was investigated to remove any soluble un-crosslinked fraction of the hydrogel discs. Therefore, the Soxhlet extraction process was carried out where unwashed dried discs of hydrogel were added into Soxhlet apparatus having deionized water at 85 °C for 12 h. The extracted discs of hydrogels were dried again in a vacuum oven at 40 °C until a constant weight was attained [80,81]. Sol–gel analysis was calculated by the given equations:

(3) $$\mathrm{Sol}\;\mathrm{fraction}\;\%=\frac{{\;W}_{1}-{\;W}_{2}}{{\;W}_{2}}\times 100$$

(4) Gel fraction=100−Sol fraction

W_1_ = initial weight of hydrogels, and W_2_ = final weight of dried hydrogels.

### 3.9. Loading of Drug

Loading of DS by hydrogel discs was performed by the dry weight method [82]. Eight-millimeter dried hydrogel discs were immersed in the drug solution of 0.8% (phosphate buffer pH 7.4) for 72 h at 25 °C. The solvent for the drug was chosen on the basis of higher drug solubility and maximum hydrogels swelling in that solvent. After 72 h, the discs were taken out and washed with distilled water to remove the attached drug on the surface of the discs. Loaded discs were placed in vacuum oven at 40 °C for 7 days after initial drying for 24 h at 25 °C. The given equation was used for estimating drug loading:

Drug loaded contents = W_D_ − W_d_ (5)

where, W_D_ = weight of dried loaded disc of hydrogel, and W_d_ = weight of dried unloaded disc of hydrogel.

### 3.10. In Vitro Drug Release Analysis

A drug release study was conducted at both acidic (pH 1.2) and basic media (pH 7.4) to analyze the percent release of commercial product of DS (Cataflam) and pH-dependent release of DS from CS/CBP-co-PAA hydrogels network. Samples were placed in 900 mL solutions of pH 1.2 and 7.4 in USP dissolution apparatus-II at 37 ± 0.5 °C and 50 rpm. The dissolution medium was collected at a specific interval of time and the same quantity of fresh medium was added back to keep the sink condition constant. The collected samples were then analyzed on UV–vis-spectrophotometer (UV-1601 Shimadzu) at 260 nm [83,84]. Kinetic modeling such as the Korsmeyer–Peppas model was carried out for all the developed formulations of CS/CBP-co-PAA hydrogels to understand the drug release mechanism from the hydrogels network [85].

## 4. Conclusions

CS/CBP-co-PAA hydrogels were successfully prepared by free radical polymerization method. SEM confirmed the porous surface of the developed hydrogels. TGA and DSC indicated the higher thermal stability of the fabricated hydrogels. PXRD revealed the successful encapsulation and reduction of the drug crystallinity by the developed polymeric network of hydrogels. FTIR proved the grafting of AA on the backbone of the CS and CBP. The swelling behavior and drug release rate were found to be very low at pH 1.2 and high at pH 7.4, indicating the pH-dependent nature of the developed hydrogels. Similarly, maximum drug release was observed from the developed system compared to the commercially available product cataflam. Keeping in view all the above results indicates that the developed hydrogel network could be used as a suitable drug carrier system for other NSAIDs too. The developed hydrogels are employed only for controlled delivery of hydrophilic drugs. Hence, in future, the development of such hydrogels is needed that could be used for multiple purposes.

## Figures and Tables

**Table 1:** Drug loading, percent drug release and kinetic modeling of CS/CBP-co-PAA interpenetrating network of hydrogels.

Formulation	Drug Loading	Percent Drug Release (48 h)		Korsmeyer-Peppas Model	
Code	(mg)	pH 1.2	pH 7.4	r2	n
CCF-1	87.01 ± 0.98	17.58 ± 0.13	92.57 ± 0.21	0.9838	0.5213
CCF-2	92.32 ± 0.70	18.06 ± 0.21	93.08 ± 0.13	0.9954	0.5978
CCF-3	95.10 ± 1.10	21.22 ± 0.32	95.40 ± 0.52	0.9909	0.5832
CCF-4	86.02 ± 0.87	23.01 ± 0.22	98.52 ± 0.19	0.9772	0.6268
CCF-5	91.21 ± 1.23	22.85 ± 0.28	96.92 ± 0.33	0.9804	0.5688
CCF-6	95.10 ± 1.10	21.22 ± 0.32	95.40 ± 0.52	0.9909	0.5832
CCF-7	89.51 ± 1.04	20.93 ± 0.30	93.10 ± 0.31	0.9871	0.6710
CCF-8	95.10 ± 1.10	21.22 ± 0.32	95.40 ± 0.52	0.9909	0.5832
CCF-9	97.24 ± 1.12	23.31 ± 0.24	99.16 ± 0.42	0.9845	0.7304

**Table 2:** Feed ratio scheme for formulation of CS/CBP-co-PAA interpenetrating polymer network (IPN) hydrogels.

Formulation Code	Polymer (CS) g/100 g	Polymer (CBP) g/100 g	Monomer (AA) g/100 g	Initiator (APS) g/100 g	Cross-Linker (EGDMA) g/100 g
CCF-1	0.160	0.640	20	0.4	1.2
CCF-2	0.320	0.640	20	0.4	1.2
CCF-3	0.480	0.640	20	0.4	1.2
CCF-4	0.480	0.320	20	0.4	1.2
CCF-5	0.480	0.480	20	0.4	1.2
CCF-6	0.480	0.640	20	0.4	1.2
CCF-7	0.480	0.640	16	0.4	1.2
CCF-8	0.480	0.640	20	0.4	1.2
CCF-9	0.480	0.640	24	0.4	1.2

## Data Availability

Not applicable.

## References

[1] Hoare T.R., Kohane D.S. (2008). Hydrogels in drug delivery: Progress and challenges. Polymer.

[2] Hennink W.E., Van Nostrum C.F. (2012). Novel crosslinking methods to design hydrogels. Adv. Drug Deliv. Rev..

[3] Suhail M., Rosenholm J.M., Minhas M.U., Badshah S.F., Naeem A., Khan K.U., Fahad M. (2019). Nanogels as drug-delivery systems: A comprehensive overview. Ther. Deliv..

[4] Buwalda S.J., Boere K.W., Dijkstra P.J., Feijen J., Vermonden T., Hennink W.E. (2014). Hydrogels in a historical perspective: From simple networks to smart materials. J. Control. Release.

[5] Loo Y., Hauser C.A. (2015). Bioprinting synthetic self-assembling peptide hydrogels for biomedical applications. Biomed. Mater..

[6] Hamidi M., Azadi A., Rafiei P. (2008). Hydrogel nanoparticles in drug delivery. Adv. Drug Deliv. Rev..

[7] Maitz M.F., Freudenberg U., Tsurkan M.V., Fischer M., Beyrich T., Werner C. (2013). Bio-responsive polymer hydrogels homeostatically regulate blood coagulation. Nat. Commun..

[8] Yan B., Boyer J.C., Habault D., Branda N.R., Zhao Y. (2012). Near infrared light triggered release of biomacromolecules from hydrogels loaded with upconversion nanoparticles. J. Am. Chem. Soc..

[9] Tomatsu I., Peng K., Kros A. (2011). Photoresponsive hydrogels for biomedical applications. Adv. Drug Deliv. Rev..

[10] Burek M., Czuba Z.P., Waskiewicz S. (2014). Novel acid-degradable and thermo-sensitive poly(N-isopropylacrylamide) hydrogels cross-linked by alpha,alpha-trehalose diacetals. Polymer.

[11] Zhao C.W., Zhuang X.L., He P., Xiao C.S., He C.L., Sun J.R., Chen X.S., Jing X.B. (2009). Synthesis of biodegradable thermo- and pH-responsive hydrogels for controlled drug release. Polymer.

[12] Nemethy A., Solti K., Kiss L., Gyarmati B., Deli M.A., Csanyi E., Szilagyi A. (2013). pH- and temperature-responsive poly(aspartic acid)-l-poly (N-isopropylacrylamide) conetwork hydrogel. Eur. Polym. J..

[13] Gyarmati B., Nemethy A., Szilagyi A. (2014). Reversible response of poly(aspartic acid) hydrogels to external redox and pH stimuli. Rsc Adv..

[14] Peppas N.A., Hilt J.Z., Khademhosseini A., Langer R. (2006). Hydrogels in biology and medicine: From molecular principles to bionanotechnology. Adv. Mater..

[15] Bhattarai N., Gunn J., Zhang M.Q. (2010). Chitosan-based hydrogels for controlled, localized drug delivery. Adv. Drug Deliv. Rev..

[16] Sperling L.H. (2012). Interpenetrating Polymer Networks and Related Materials.

[17] Rao K.S.V.K., Naidu B.V.K., Subha M.C.S., Sairam M., Aminabhavi T.M. (2006). Novel chitosan-based pH-sensitive interpenetrating network microgels for the controlled release of cefadroxil. Carbohydr. Polym..

[18] Kulkarni R.V., Sreedhar V., Mutalik S., Setty C.M., Sa B. (2010). Interpenetrating network hydrogel membranes of sodium alginate and poly(vinyl alcohol) for controlled release of prazosin hydrochloride through skin. Int. J. Biol. Macromol..

[19] Pescosolido L., Vermonden T., Malda J., Censi R., Dhert W.J.A., Alhaique F., Hennink W.E., Matricardi P. (2011). In situ forming IPN hydrogels of calcium alginate and dextran-HEMA for biomedical applications. Acta Biomater..

[20] Rubinstein A., Nakar D., Sintov A. (1992). Chondroitin Sulfate—A Potential Biodegradable Carrier for Colon-Specific Drug Delivery. Int. J. Pharm..

[21] Morris J.D., Smith K.M. (2009). Chondroitin Sulfate in Osteoarthritis Therapy. Orthopedics.

[22] Richy F., Bruyere O., Ethgen O., Rabenda V., Bouvenot G., Audran M., Cuenca G.H.B., Moore A., Eliakim R., Van De Putte L. (2003). Time-dependent risk of gastrointestinal complications induced by NSAIDS use: A consensus statement using meta-analytic approach. Nat. Mater..

[23] Wang D.A., Varghese S., Sharma B., Strehin I., Fermanian S., Gorham J., Fairbrother D.H., Cascio B., Elisseeff J.H. (2007). Multifunctional chondroitin sulphate for cartilage tissue-biomaterial integration. Nat. Mater..

[24] Li L.L., Mathias N.R., Heran C.L., Moench P., Wall D.A., Smith R.L. (2006). Carbopol-mediated paracellular transport enhancement in Calu-3 cell layers. J. Pharm. Sci..

[25] Gu J.M., Robinson J.R., Leung S.H.S. (1988). Binding of Acrylic Polymers to Mucin Epithelial Surfaces—Structure-Property Relationships. Crit. Rev. Ther. Drug Carrier Syst..

[26] Kabiri K., Lashani S., Zohuriaan-Mehr M.J., Kheirabadi M. (2011). Super alcohol-absorbent gels of sulfonic acid-contained poly(acrylic acid). J. Polym. Res..

[27] Barry B.W., Meyer M.C. (1979). Rheological Properties of Carbopol Gels 1. Continuous Shear and Creep-Properties of Carbopol Gels. Int. J. Pharm..

[28] Fresno M.J., Ramirez A.D., Jimenez M.M. (2002). Systematic study of the flow behaviour and mechanical properties of Carbopol Ultrez 10 hydroalcoholic gels. Eur. J. Pharm. Biopharm..

[29] Pooley S.A., Rivas B.L., Lillo F.E., Pizarro G.D. (2010). Hydrogels from Acrylic Acid with N,N-Dimethylacrylamide: Synthesis, Characterization, and Water Absorption Properties. J. Chil. Chem. Soc..

[30] Amin M.C.I.M., Ahmad N., Halib N., Ahmad I. (2012). Synthesis and characterization of thermo- and pH-responsive bacterial cellulose/acrylic acid hydrogels for drug delivery. Carbohydr. Polym..

[31] Sena M.M., Chaudhry Z.F., Collins C.H., Poppi R.J. (2004). Direct determination of diclofenac in pharmaceutical formulations containing B vitamins by using UV spectrophotometry and partial least squares regression. J. Pharm. Biomed. Anal..

[32] Sweetman S.C. (2009). Martindale: The Complete Drug Reference.

[33] Arias J.L., Lopez-Viota M., Lopez-Viota J., Delgado A.V. (2009). Development of iron/ethylcellulose (core/shell) nanoparticles loaded with diclofenac sodium for arthritis treatment. Int. J. Pharm..

[34] Italia J.L., Bhatt D.K., Bhardwaj V., Tikoo K., Kumar M.N. (2007). PLGA nanoparticles for oral delivery of cyclosporine: Nephrotoxicity and pharmacokinetic studies in comparison to Sandimmune Neoral. J. Control. Release.

[35] Italia J.L., Yahya M.M., Singh D., Kumar M.N.V.R. (2009). Biodegradable Nanoparticles Improve Oral Bioavailability of Amphotericin B and Show Reduced Nephrotoxicity Compared to Intravenous FungizoneA (R). Pharm. Res..

[36] Khanum H., Ullah K., Murtaza G., Khan S.A. (2018). Fabrication and in vitro characterization of HPMC-g-poly(AMPS) hydrogels loaded with loxoprofen sodium. Int. J. Biol. Macromol..

[37] Peppas N.A., Sahlin J.J. (1989). A Simple Equation for the Description of Solute Release. 3. Coupling of Diffusion and Relaxation. Int. J. Pharm..

[38] Naidu V.G.M., Madhusudhana K., Sashidhar R.B., Ramakrishna S., Khar R.K., Ahmed F.J., Diwan P.V. (2009). Polyelectrolyte complexes of gum kondagogu and chitosan, as diclofenac carriers. Carbohydr. Polym..

[39] Wang L.F., Shen S.S., Lu S.C. (2003). Synthesis and characterization of chondroitin sulfate-methacrylate hydrogels. Carbohydr. Polym..

[40] Loh G.O.K., Tan Y.T.F., Peh K.K. (2014). Hydrophilic polymer solubilization on norfloxacin solubility in preparation of solid dispersion. Powder Technol..

[41] Shen X., Yu D., Zhu L., Branford-White C., White K., Chatterton N.P. (2011). Electrospun diclofenac sodium loaded Eudragit(R) L 100-55 nanofibers for colon-targeted drug delivery. Int. J. Pharm..

[42] Amrutkar J.R., Gattani S.G. (2009). Chitosan-chondroitin sulfate based matrix tablets for colon specific delivery of indomethacin. AAPS PharmSciTech.

[43] Lee W.F., Chiang W.H. (2004). Swelling and drug-release behavior of the poly(AA-co-N-vinyl pyrrolidone)/chitosan interpenetrating polymer network hydrogels. J. Appl. Polym. Sci..

[44] Singh B., Dhiman A. (2019). Functionalization of carbopol with NVP for designing antibiotic drug loaded hydrogel dressings for better wound management. J. Pharm. Biopharm. Res..

[45] Sarfraz R.M., Khan M.U., Mahmood A., Akram M.R., Minhas M.U., Qaisar M.N., Ali M.R., Ahmad H., Zaman M. (2020). Synthesis of co-polymeric network of carbopol-g-methacrylic acid nanogels drug carrier system for gastro-protective delivery of ketoprofen and its evaluation. Polym. Plast. Technol. Mater..

[46] Shah R., Saha N., Saha P. (2015). Influence of temperature, pH and simulated biological solutions on swelling and structural properties of biomineralized (CaCO3) PVP-CMC hydrogel. Prog. Biomater..

[47] Agnihotri S.M., Vavia P.R. (2009). Diclofenac-loaded biopolymeric nanosuspensions for ophthalmic application. Nanomedicine.

[48] Swain R.P., Nagamani R., Panda S. (2015). Formulation, in vitro characterization and stability studies of fast dispersing tablets of diclofenac sodium. J. Appl. Pharm. Sci..

[49] Crispim E.G., Piai J.F., Fajardo A.R., Ramos E.R.F., Nakamura T.U., Nakamura C.V., Rubira A.F., Muniz E.C. (2012). Hydrogels based on chemically modified poly(vinyl alcohol) (PVA-GMA) and PVA-GMA/chondroitin sulfate: Preparation and characterization. Express Polym. Lett..

[50] Khalid I., Ahmad M., Minhas M.U., Barkat K. (2018). Synthesis and evaluation of chondroitin sulfate based hydrogels of loxoprofen with adjustable properties as controlled release carriers. Carbohydr. Polym..

[51] Sahoo S., Chakraborti C.K., Mishra S.C. (2011). Qualitative analysis of controlled release ciprofloxacin/carbopol 934 mucoadhesive suspension. J. Adv. Pharm. Technol. Res..

[52] Sahoo S., Chakraborti C.K., Mishra S.C. (2011). Antibacterial activity study of a mucoadhesive suspension containing ciprofloxacin. Afr. J. Microbiol. Res..

[53] Patel M.P., Patel R.R., Patel J.K. (2010). Chitosan mediated targeted drug delivery system: A review. J. Pharm. Pharm. Sci..

[54] Moharram M.A., Khafagi M.G. (2007). Application of FTIR spectroscopy for structural characterization of ternary poly(acrylic acid)-metal-poly(vinyl pyrrolidone) complexes. J. Appl. Polym. Sci..

[55] Khalid I., Ahmad M., Minhas M., Barkat K., Sohail M. (2018). Cross-Linked Sodium Alginate-g-poly(Acrylic Acid) Structure: A Potential Hydrogel Network for Controlled Delivery of Loxoprofen Sodium. Adv. Polym. Technol..

[56] Sohail M., Ahmad M., Minhas M.U., Ali L., Munir A., Khalid I. (2014). Synthesis and Characterization of Graft PVA Composites for Controlled Delivery of Valsartan. Lat. Am. J. Pharm..

[57] Barkat K., Ahmad M., Minhas M.U., Khalid I. (2017). Oxaliplatin-loaded crosslinked polymeric network of chondroitin sulfate-co-poly(methacrylic acid) for colorectal cancer: Its toxicological evaluation. J. Appl. Polym. Sci..

[58] Sharmin N., Elias-Al-Mamun M., Jalil R.U. (2010). A novel method to study the effect of pH and excipients on water uptake and swelling behaviour of carbopol polymers. Bangladesh Pharm. J..

[59] Sullad A.G., Manjeshwar L.S., Aminabhavi T.M. (2010). Novel pH-Sensitive Hydrogels Prepared from the Blends of Poly(vinyl alcohol) with Acrylic Acid-graft-Guar Gum Matrixes for Isoniazid Delivery. Ind. Eng. Chem. Res..

[60] Harish N.M., Prabhu P., Charyulu R.N., Gulzar M.A., Subrahmanyam E.V. (2009). Formulation and Evaluation of in situ Gels Containing Clotrimazole for Oral Candidiasis. Indian J. Pharm. Sci..

[61] Nasir N., Ahmad M., Minhas M.U., Barkat K., Khalid M.F. (2019). pH-responsive smart gels of block copolymer [pluronic F127-co-poly(acrylic acid)] for controlled delivery of Ivabradine hydrochloride: Its toxicological evaluation. J. Polym. Res..

[62] Dergunov S.A., Nam I.K., Mun G.A., Nurkeeva Z.S., Shaikhutdinov E.M. (2005). Radiation synthesis and characterization of stimuli-sensitive chitosan-polyvinyl pyrrolidone hydrogels. Radiat. Phys. Chem..

[63] Murthy P.S.K., Mohan Y.M., Sreeramulu J., Raju K.M. (2006). Semi-IPNs of starch and poly(acrylamide-co-sodium methacrylate): Preparation, swelling and diffusion characteristics evaluation. React. Funct. Polym..

[64] Rashid H., Ahmad M., Minhas M.U., Sohail M., Aamir M.F. (2015). Synthesis and Characterization of Poly(hydroxyethyl methacrylate-co-methacrylic acid) Cross Linked Polymeric Network for the Delivery of Analgesic Agent. J. Chem. Soc. Pak..

[65] Oprea A.M., Ciolacu D., Neamtu A., Mungiu O.C., Stoica B., Vasile C. (2010). Cellulose/Chondroitin Sulfate Hydrogels: Synthesis, Drug Loading/Release Properties and Biocompatibility. Cell. Chem. Technol..

[66] Khan G.M., Jiabi Z. (1998). Formulation and in vitro evaluation of ibuprofen-carbopol (R) 974P-NF controlled release matrix tablets III: Influence of co-excipients on release rate of the drug. J. Control. Release.

[67] Khan G.M., Zhu J.B. (1999). Studies on drug release kinetics from ibuprofen-carbomer hydrophilic matrix tablets: Influence of co-excipients on release rate of the drug. J. Control. Release.

[68] Sanli O., Ay N., Isiklan N. (2007). Release characteristics of diclofenac sodium from poly(vinyl alcohol)/sodium alginate and poly(vinyl alcohol)-grafted-poly(acrylamide)/sodium alginate blend beads. Eur. J. Pharm. Biopharm..

[69] Shoaib M.H., Tazeen J., Merchant H.A., Yousuf R.I. (2006). Evaluation of drug release kinetics from ibuprofen matrix tablets using HPMC. Pak. J. Pharm. Sci..

[70] Bernardi A., Zilberstein A.C., Jager E., Campos M.M., Morrone F.B., Calixto J.B., Pohlmann A.R., Guterres S.S., Battastini A.M. (2009). Effects of indomethacin-loaded nanocapsules in experimental models of inflammation in rats. Br. J. Pharmacol..

[71] Suhail M., Wu P.-C., Minhas M.U. (2020). Using Carbomer-Based Hydrogels for Control the Release Rate of Diclofenac Sodium: Preparation and In Vitro Evaluation. Pharmaceuticals.

[72] Sarfraz R.M., Khan H.U., Mahmood A., Ahmad M., Maheen S., Sher M. (2015). Formulation and evaluation of mouth disintegrating tablets of atenolol and atorvastatin. Indian J. Pharm. Sci..

[73] Suhail M., Wu P.-C., Minhas M.U. (2021). Development and characterization of pH-sensitive chondroitin sulfate-co-poly (acrylic acid) hydrogels for controlled release of diclofenac sodium. J. Saudi Chem. Soc..

[74] Mahmood A., Ahmad M., Sarfraz R.M., Minhas M.U., Yaqoob A. (2016). Formulation and in Vitro Evaluation of Acyclovir Loaded Polymeric Microparticles: A Solubility Enhancement Study. Acta Pol. Pharm..

[75] Suhail M., Khan A., Rosenholm J.M., Minhas M.U., Wu P.-C. (2021). Fabrication and Characterization of Diclofenac Sodium Loaded Hydrogels of Sodium Alginate as Sustained Release Carrier. Gels.

[76] Mahmood A., Ahmad M., Sarfraz R.M., Minhas M.U. (2018). Development of Acyclovir Loaded -Cyclodextrin-g-Poly Methacrylic Acid Hydrogel Microparticles: An In Vitro Characterization. Adv. Polym. Technol..

[77] Suhail M., Fang C.-W., Minhas M.U., Wu P.-C. (2021). Preparation, Characterization, Swelling Potential and In-Vitro Evaluation of Sodium Poly (Styrene Sulfonate)-Based Hydrogels for Controlled Delivery of Ketorolac Tromethamine. Pharmaceuticals.

[78] Khalid I., Ahmad M., Minhas M.U., Barkat K. (2018). Preparation and characterization of alginate-PVA-based semi-IPN: Controlled release pH-responsive composites. Polym. Bull..

[79] Zahra Q., Minhas M.U., Khan S., Wu P.-C., Suhail M., Iqbal R., Bashir M. (2021). Fabrication of polyethylene glycol hydrogels with enhanced swelling; loading capacity and release kinetics. Polym. Bull..

[80] Singh B., Sharma N. (2009). Mechanistic Implication for Cross-Linking in Sterculia-Based Hydrogels and Their Use in GIT Drug Delivery. Biomacromolecules.

[81] Khan K.U., Minhas M.U., Sohail M., Badshah S.F., Abdullah O., Khan S., Munir A., Suhail M. (2021). Synthesis of PEG-4000-co-poly (AMPS) nanogels by cross-linking polymerization as highly responsive networks for enhancement in meloxicam solubility. Drug Dev. Ind. Pharm..

[82] Sohail M., Ahmad M., Minhas M.U., Ali L., Khalid I., Rashid H. (2015). Controlled delivery of valsartan by cross-linked polymeric matrices: Synthesis, in vitro and in vivo evaluation. Int. J. Pharm..

[83] Bukhari S.M.H., Khan S., Rehanullah M., Ranjha N.M. (2015). Synthesis and Characterization of Chemically Cross-Linked Acrylic Acid/Gelatin Hydrogels: Effect of pH and Composition on Swelling and Drug Release. Int. J. Polym. Sci..

[84] Suhail M., Hsieh Y.-H., Khan A., Minhas M.U., Wu P.-C. (2021). Preparation and In Vitro Evaluation of Aspartic/Alginic Acid Based Semi-Interpenetrating Network Hydrogels for Controlled Release of Ibuprofen. Gels.

[85] Yu J., Warnke J., Lyubchenko Y.L. (2011). Nanoprobing of alpha-synuclein misfolding and aggregation with atomic force microscopy. Nanomedicine.

